# Near-Infrared Sub-Bandgap All-Silicon Photodetectors: State of the Art and Perspectives

**DOI:** 10.3390/s101210571

**Published:** 2010-11-29

**Authors:** Maurizio Casalino, Giuseppe Coppola, Mario Iodice, Ivo Rendina, Luigi Sirleto

**Affiliations:** Istituto per la Microelettronica e Microsistemi (IMM), Consiglio Nazionale delle Ricerche (CNR), Via P. Castellino 111, 80131 Napoli, Italy; E-Mails: giuseppe.coppola@na.imm.cnr.it (G.C.); mario.iodice@na.imm.cnr.it (M.I.); ivo.rendina@na.imm.cnr.it (I.R.); luigi.sirleto@na.imm.cnr.it (L.S.)

**Keywords:** optoelectronics, photodetector, silicon, waveguide, ring resonator, absorption

## Abstract

Due to recent breakthroughs, silicon photonics is now the most active discipline within the field of integrated optics and, at the same time, a present reality with commercial products available on the market. Silicon photodiodes are excellent detectors at visible wavelengths, but the development of high-performance photodetectors on silicon CMOS platforms at wavelengths of interest for telecommunications has remained an imperative but unaccomplished task so far. In recent years, however, a number of near-infrared all-silicon photodetectors have been proposed and demonstrated for optical interconnect and power-monitoring applications. In this paper, a review of the state of the art is presented. Devices based on mid-bandgap absorption, surface-state absorption, internal photoemission absorption and two-photon absorption are reported, their working principles elucidated and their performance discussed and compared.

## 1. Introduction

Silicon photonics is a technology for implementing various optical functionalities in silicon, offering the potential for large-scale integration of multiple optical and electronic functions on a single silicon chip. This should result in revolutionizing applications in microelectronics (through the replacement of metal/dielectric stacks with optical interconnects), in telecommunications (integrated optical circuits), and in biological and chemical sensing. Significant advantages exist when using silicon as the base material for such devices: in particular, the vast amount of research now available to designers and process engineers on all aspects of the material system, as well as the established, global silicon processing industry that has evolved from almost six decades of silicon-based microelectronics fabrication. Silicon wafers have the lowest cost per unit area and the highest crystal quality of any semiconductor material. The industry is able to produce microprocessors with hundreds of millions of components—all integrated onto thumb-sized chips—and offers them at such low prices that they are increasingly used in consumer electronics. Silicon manufacturing represents the most spectacular convergence of technological sophistication and economics of scale [1–3].

Silicon photonics was pioneered by Soref during the 1980 [4–7]. Creating low-cost photonics for mass-market applications by exploiting the powerful IC industry has been the traditional motivation of silicon photonics researchers. Silicon is used in photonics due to its good optical properties, the low cost of the material and its easy manufacturability; at present, however, it is not suitable for the manufacture of active devices. Despite the success of hybrid technologies in achieving active optical components, many people think that only all-silicon active devices will make silicon photonics a “killer technology”. Following this line of reasoning, tremendous progress has recently been made in technological processes based on the use of silicon-on-insulator (SOI) substrates, which has permitted the obtention of reliable and effective fully CMOS-compatible optical components such as low-loss waveguides, high-Q resonators, high-speed modulators, couplers, and optically pumped lasers. All these devices have been developed to operate in the wavelength range from C band (1,528–1,561 nm) to L band (1,561–1,620 nm) where defect-free intrinsic bulk silicon has minimal absorption [8–13].

One of the crucial steps toward the integration of microphotonics with silicon-based electronics is the development of efficient chip-scale photodetectors (PD) integrated on silicon. Bulk photodetectors are perhaps the oldest and best understood silicon optoelectronic devices. Commercial products operate at wavelengths below 1,100 nm, where band-to-band absorption occurs. Additionally, they are used in conjunction with scintillators, and are widely used as x-ray detectors applied in medical tomography equipment and airport luggage scanners. One of the most important advantages of silicon is that, due to the quality of its crystalline material and its excellent passivation properties, very low dark-current PD devices can be obtained [14].

For the realization of photodiodes integrated in photonics circuits operating at wavelengths beyond 1,100 nm, silicon was not considered the right material because of its transparency. However, in recent years, in order to take advantage of low-cost standard Si-CMOS processing technology, a number of photodetectors have been proposed based on different physical effects, such as: mid-bandgap absorption (MBA), surface-state absorption (SSA), internal photoemission absorption (IPA) and two-photon absorption (TPA).

In this paper, an overview of the state of the art on near-IR (NIR) sub-bandgap all-silicon photodetectors is presented. First, the physical effects and the working principles of these devices are elucidated. Then, the main structures reported in the literature and the most significant results obtained in recent years are reviewed and discussed, comparing the performance of devices based on different approaches.

## 2. Photodetector Performance Requirements

It is useful to briefly recall the main performance requirements of integrated photodetectors for NIR optical applications. High speed, high responsivity, low dark current, low bias voltage and small dimensions are appealing properties for a photodetector and research efforts are now aimed at achieving all these in single devices.

As bandwidth demand keeps increasing, it is essential that all-silicon photodetectors operate at 5 GHz, a frequency at which hybrid detectors have already been demonstrated [15]. Of course, higher bandwidths (20 GHz, >50 GHz) are desirable in order to anticipate future trends in optical interconnects. In fact, very high-speed photodetectors, combined with dense wavelength division multiplexing (DWDM) technology in the C band (1,528–1,561 nm) and L band (1,561–1,620 nm) [16], have the potential to achieve a bandwidth greater than 0.5 THz.

Another important property of a detector is described by its responsivity, which indicates the current produced by a certain optical power. Reasonable responsivities are necessary for an acceptable signal-to-noise ratio and to ease the design and realization of the amplifier circuitry that follows. Responsivity is strictly linked to a device’s quantum efficiency, a property describing how many carriers per photon are collected. It is worth noting that there is a difference between internal and external quantum efficiency: in the case of internal quantum efficiency, the number of carriers that contribute to the photocurrent is related to the number of absorbed photons, while in the case of external quantum efficiency, they are related to the number of incident photons. In the telecommunications field a responsivity ≥ 0.1 A/W [17], corresponding to external quantum efficiencies η of 15%, 10%, and 8% at λ = 850, 1,300, and 1,550 nm, respectively, is required.

In order to evaluate detector performance, an important property is dark current. This is a serious issue because the shot noise, associated with the fluctuations in a measured signal due to the random arrival time of the particles carrying energy, generates a leakage current which can increase the bit error rate (BER). Dark current depends on work frequency and it is worth noting that a higher dark current could be allowed if a system worked at frequencies at which the amplifier noise overcame photodetector noise. In a typical photodetector, dark currents less than 1 μA are required.

A further requirement of photodetectors is low-voltage operation. It would be desirable to realize devices operating at the same power supply as the CMOS circuitry, *i.e.*, bias voltage < 5 V and as low as 1 V for advanced CMOS generation.

Finally, the shrinking of photodetector dimensions would allow for the integration of photonic components with integrated electronic circuits, enabling interconnection bandwidths that are not limited by the RC time constant or the reliability constraints of metal lines.

## 3. Near-Infrared Silicon Absorption Physics

In order to develop NIR all-silicon photodetectors while taking advantage of low-cost standard silicon processing technology without additional material or process steps, a number of options have been proposed. In this paragraph, in order to elucidate the physical effects behind the working principles of recently proposed devices, we introduce photoconductivity phenomena in the first paragraph, while two-photon absorption is reported in the last paragraph.

### 3.1. Photoconductivity and Linear Absorption

The term photoconductivity covers all the phenomena by which a change in conductivity—either an increase or decrease—follows absorption of light in the considered materials. Photoconductivity is not an elementary process. It includes several successive or simultaneous mechanisms: optical absorption, hot carrier relaxation, charge carrier transport and recombination. Photoconductivity offers a means of studying many physical properties of materials and, on the other hand, photoconductivity effects have obvious applications for the detection and measurement of light over the whole electromagnetic spectrum. The corresponding literature is very abundant and can be found in many scientific journals and books. Therefore, in this paragraph we don’t intend to review all the published papers dealing with photoconductivity and related effects, but instead to provide a short introduction to the physical effects behind the working principles of recently proposed near-infrared all-silicon photodetectors [18].

The observation of any photoconductivity phenomenon requires the presence of at least one type of mobile charge carrier. Photoconductivity is due to a change in carrier concentration, which results from the generation in the semiconductor of electronic excited states by absorption of light energy. The excited states have a finite lifetime: they lose energy through different processes of relaxation and recombination. Finally, we note that in homogeneous solids, photoconductivity is usually observed by applying an electric field to the material and measuring the resulting photocurrent.

One of the most important aspects is the generation of photocurrent, which is obtained by irradiating a sample with a homogeneous light flux. Inter-band generation, which is due to optical absorption by inter-band electronic transition, is characterized by high absorption coefficients. Intra-band generation refers to free-carrier optical absorption. In this case, the absorption coefficient is usually low and is proportional to the free-carrier concentration. Finally, we have extrinsic generation, which refers to photoionization of impurities and localized centers. There are two main differences with respect to the case of inter-band generation: only one excited carrier is generated, either electron or hole, and the absorption process is characterised by low absorption coefficients [19,20]. Photoconductivity effects related to extrinsic generation have been recently investigated in order to realize NIR silicon photodetectors. Therefore, in the next two subsections, the photoconductivity effect due to mid-bandgap absorption (MBA), and due to surface state absorption (SSA), is reported. Finally, in the last subsection, internal photoemission absorption (IPA) is discussed.

#### 3.1.1. Mid-Bandgap Absorption (MBA)

Defects always exist in semiconductors. They may be extrinsic, involving foreign atoms (*i.e*., impurities) or intrinsic due to crystalline defects. Impurities can be intentionally introduced as dopant or recombination centers in order to make a semiconductor useful for fabricating a device, or they can be unintentionally incorporated during crystal growth or device processing, with undesirable effects. By crystalline defect, one generally means any region where the microscopic arrangement of atoms differs drastically from that of a perfect crystal. Defects are classified into point defects, line defects and cluster defects. Among the most important kind of defects we mention: vacancies and interstitials (point defects), dislocations (line defects) [21,22], divacancies and interstitial clusters (cluster defects) [23].

It is well known that the band diagram of a perfect crystal semiconductor consists of a valence band and a conduction band separated by the band gap, with no energy levels within the band gap. When the periodicity of the single crystal is perturbed by foreign atoms or crystal defects, discrete energy levels are introduced into the band gap. Usually, we can separate defects in two broad categories: shallow impurities and deep centers. The former are characterized by levels with binding energies much smaller than the energy gap of the host semiconductor, while the latter are characterized by electronic levels located near the middle of the bandgap. The simplest type of shallow impurity is substitutional. In an energy level diagram, an electron from a substitutional impurity lies closer to the conduction band than a valence electron. A photon whose energy is at least equal to the energy difference can elevate an electron from an impurity level to the conduction band. The ionization energy is a few meV, permitting the absorption in the far-infrared regime. Another simple case is that of interstitial impurities, which produce a second type of optical effect: impurity changes in the local lattice environment, resulting in an alteration of vibrational behaviour in the atoms located in the neighbourhood of each impurity. This can lead to new infrared vibrational modes [21,22].

Defects called generation-recombination centers lie deep in the band gap. They act as recombination centers when there are excess carriers in the semiconductor, and as generation centers when carrier density is below its equilibrium value. For single crystal semiconductors, like silicon and germanium, deep level impurities are usually metallic impurities, but they can be crystal imperfections, such as dislocations, vacancies or interstitials. For the most part, they are undesirable. Occasionally, however, they are deliberately introduced to alter a device characteristic.

Absorption of light is one of the most successful methods for investigating the impurity electronic state. The simplest information which can be obtained from absorption measurements is the energetic position of the impurity level within the forbidden energy gap of the semiconductor. This process can also be observed using photoconductivity measurements in bulk materials and photocurrent measurements on p-n as well as Schottky junctions. Extrinsic photoconductivity offers a convenient means of detecting and studying impurities and localized centers. Also, by changing the type of impurity in a given material, the spectral sensitivity can be adjusted over a wide wavelength range. Obvious applications for infrared detection exist and encourage a strong interest in this field [18–20].

**Figure 1.**
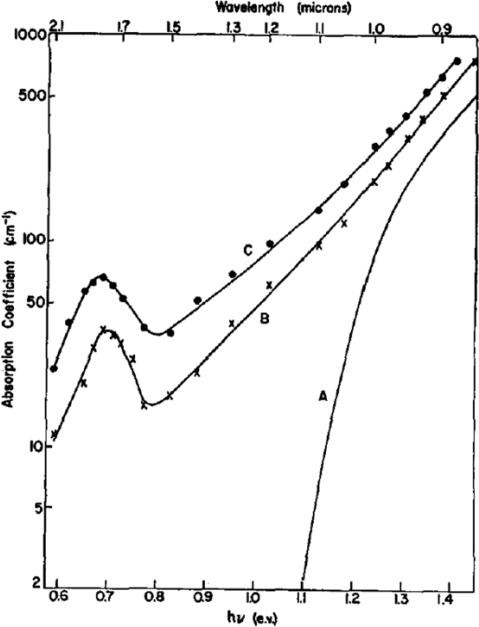
Absorption spectrum of deuteron-irradiated silicon. A: normal silicon; B and C: after successive deuteron irradiations [24]. Copyright 1959, American Institute of Physics.

As an example of optical absorption due to defect introduction, it is interesting to report the experimental results carried out by Fan and Ramdas in 1959 [24]. In Figure 1, curve A shows the intrinsic absorption edge in un-implanted high-resistivity silicon; curves B and C are two of the curves obtained on a sample after successive deuteron irradiations.

Due to the irradiation effect at wavelengths less than 1 μm, an apparent shift of the absorption edge toward lower energy can be noted (Figure 1). The shift could be caused by a modification of the band structure due to the presence of defects. Under prolonged irradiation, the effect saturates after a shift of about 0.1 eV. The sub-bandgap absorption in the irradiated sample is possible at photon energy as low as 0.41 eV, *i.e.*, at wavelengths up to 3 μm.

Starting from these results, many authors have induced mid-bandgap energy levels via defect incorporation into the silicon lattice by Si^+^ ion implantation. For this material system, two typologies of crystal defects are believed responsible for sub-bandgap radiation absorption: divacancies and interstitial clusters [23].

#### 3.1.2. Surface-State Absorption (SSA)

The spatial distributions and the energies of the electrons (and holes) near and on the clean surface of a chemically pure and physically perfect crystalline solid in a vacuum differ from those of bulk silicon due to the interruption of the periodic potential at the surface. Near and on the surface, some of the wave functions are not localized, while others are localized or bound with discrete energies. In contrast, wave functions in the bulk materials are all spread out over the entire crystal, and the electron energies represented by these wave functions will group into bands (allowed energy bands) separated by gaps (forbidden energy gaps). Surface electronic states are further modified by the presence of foreign atoms on the solid surface, as well as from contact with a gas, liquid or solid. The electronic states on a clean surface in a vacuum are known as surface states, while those on a surface in contact with another material are known as interface states. In addition, the electronic states may be intrinsic, owing to the displacement of the host atoms of the two materials, making the contact or forming the interface—or they may be extrinsic, due to the presence of foreign atoms or molecules. Finally, we note that—since the bulk and the surface of a crystal have distinct band structures, and the solid, considered as an isolated system, must have a constant Fermi level—there is an exchange of electrons between the surface and the bulk, which, in the case of a semiconductor, generates a space charge region close to the surface [25].

Among the oxide semiconductor systems, the SiO_2_-Si system is the most important one. There are four general types of trapped charges: fixed oxide charge, mobile oxide charge, oxide-trapped charge and interfacial-trapped charge [26]. They are described as follows:

***Interface-trapped charge***: these are positive or negative charges due to structural defects, oxidation-induced defects, metal impurities or other defects caused by radiation or similar bond-breaking processes. The interface-trapped charge is located at the Si-SiO_2_ interface and it is in electrical communication with the underlying silicon.

***Fixed Oxide Charge***: This is a positive charge due to structural defects (ionized silicon) in the oxide layer less than 2 nm from the Si-SiO_2_ interface [26]. The density of this charge, whose origin is related to the oxidation process, depends on the oxidation ambient and temperature and on the silicon substrate. This fixed oxide charge is not in electrical communication with the underlying silicon.

***Oxide-Trapped Charge:*** This charge may be positive or negative due to holes or electrons trapped in the bulk of the oxide. Trapping may result from ionizing radiation, avalanche injection and other mechanisms.

***Mobile Oxide Charge***: This is caused primarily by ionic impurities such as Na^+^, Li^+^, K^+^ and possibly H^+^. Negative ions and heavy metals may contribute to this charge.

**Figure 2.**
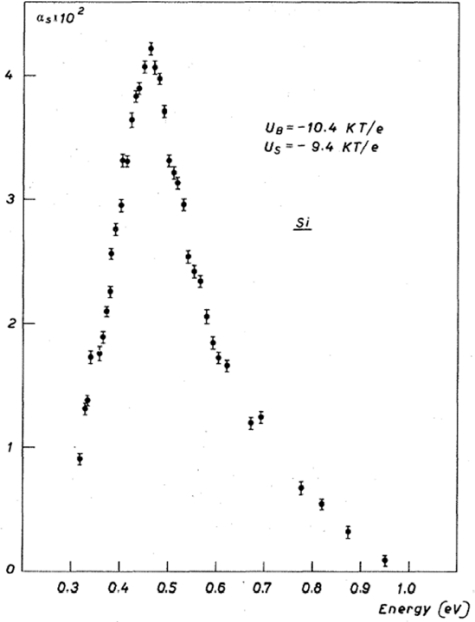
Absorption constant αs *vs.* photon energy for a silicon surface [27]. Copyright (1971) by the American Physical Society [28].

We note that surface states are known to be located within the first three or four atomic layers from the surface. It means that beyond 10 Å from the surface, electron states are bulk states. When a monochromatic photon beam interacts with a solid, electronic transitions give an absorption coefficient which can be very low when the solid is transparent, but which cannot exceed 10^−6^ cm^−1^, whatever the wavelength. This means that in the case of strongly absorbed photons, the main part of the absorption process corresponds to transitions between bulk states since the depth of penetration of the photon is at least in the 100 Å range. Therefore, one important difficulty in using optical methods to study surface states is to recognize where surface states are effectively involved in the experimental measurements. As example of optical absorption due to surface states the results obtained by Chiarotti *et al.* [27] are illustrated in Figure 2: the presence of electronic surface states, localized in the forbidden energy gap of a semiconductor, causes optical absorption extending to energies lower than the bandgap energy. Different processes could be useful to explain the results in Figure 2: (i) optical transitions between two bands of surface states localized in the gap, (ii) optical transitions from the valence band to an empty band of surface states located in the gap and (iii) optical transitions from a filled band of surface states in the gap to the empty levels of the conduction band.

#### 3.1.3. Internal Photoemission Absorption (IPA)

**Figure 3.**
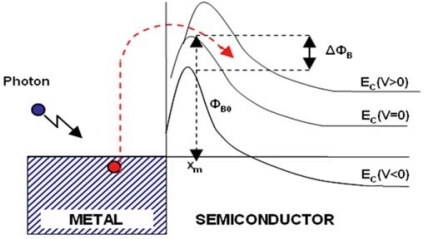
Energy band diagram for a metal/n-semiconductor junction. Reprinted with permission from M. Casalino *et al.* [29] IOP Publishing is acknowledged.

Internal photoemission is the optical excitation of electrons in the metal to energy above the Schottky barrier and then transport of these electrons to the conduction band of the semiconductor (Figure 3).

The standard theory of photoemission from a metal into a vacuum is due to Fowler [30]. It assumes that the density of states in the metal is described by the normal parabolic distribution associated with nearly-free electron approximation. Thus in Fowler’s theory, the Fermi level is several eV above the conduction band minimum when a single band is considered. In a gas of electrons obeying the Fermi-Dirac statistic, the fraction of the absorbed photons F_e_ which produces photoelectrons with the appropriate energy and momentum before scattering to contribute to the photocurrent is calculated as:

(1) $$F_{e}=\frac{\left[{\left(h\nu -(\varphi_{B0}-\Delta \varphi_{B})\right)}^{2}+\frac{{\left(k_{B}T\pi \right)}^{2}}{3}-2{\left(k_{B}T\right)}^{2}e^{\frac{h\nu -(\varphi_{B0}-\Delta \varphi_{B})}{k_{B}T}}\right]}{8k_{B}TE_{F}\text{log}\left[1+e^{\frac{h\nu -(\varphi_{B0}-\Delta \varphi_{B})}{k_{B}T}}\right]}$$

where hν is photon energy, Φ_B0_ is the potential barrier at zero bias, ΔΦ_B_ is the lowering due to image force effect (as we will see later), k is Boltzman’s constant, T is the absolute temperature and E_F_ is the metal Fermi level.

Fowler’s theory was originally obtained without taking into account the thickness of the Schottky metal layer. In order to study the quantum efficiency for thin metal films, the theory must be further extended, taking into account multiple reflections of the excited electrons from the surfaces of the metal film, in addition to collisions with phonons, imperfections and cold electrons.

Assuming a thin metal film, a phenomenological semi-classical ballistic transport model for the effects of the scattering mechanisms resulting in a multiplicative factor for quantum efficiency has been developed by Vickers [31].

The efficiency in collecting those electrons which have sufficient normal kinetic energy to overcome Φ_B_ will depend on their probability of collisions with cold or hot electrons and with the two boundary surfaces of the metal. Supposing that the Schottky barrier is illuminated through the front surface of the metal electrode, and that optical absorption is uniform throughout the metal thickness, we indicate with p_t_ the average probability that photoexcited electrons arrive at the semiconductor surface without collision. Then, we indicate with p_r_ the accumulated probability of arrival at the reflection boundary of those electrons which have sufficient energy to overcome Φ_B_ after one collision with cold or hot electrons, which are are oriented with equal probability over the entire half-sphere before reflection by the metal surface.

Finally, for multiple reflections, we indicate with p the probability that the capturable electrons can bounce from one metal boundary to another. Therefore, the total accumulated probability that the electrons will have sufficient normal kinetic energy to overcome Φ_B_ is given by [32]:

(2) $$P_{E}=p_{t}+p_{r}(1+p+p^{2}+p^{3}+\ldots )\text{exp}(-d/L)≅\frac{L}{d}{\left[1-e^{\frac{d}{L}}\right]}^{\frac{1}{2}}$$

where d is the metal thickness and L the mean free path describing the probability of collision with cold or hot electrons [32]. It would be possible to show by plotting Equation 2 that by decreasing metal thickness d, P_E_ increases, and that a considerable gain can be obtained when d is much smaller than L (thin film). This gain is due to the increased probability of emission of carriers. In a recent work [33], Scales and Berini show that a further enhancement of this probability emission can be obtained in structures realized with thin metal film buried in a semiconductor and forming two Schottky barriers.

In order to estimate the number of electrons which will be able to overcome the potential barrier, two other factors must be taken into account: image force effect and barrier collection efficiency. The image force effect between an electron and the surface of metal causes a potential barrier lowering (ΔΦ_B_) and a displacement (x_m_), as shown in Figure 3, which can be calculated with the following formula: [34].

(3) $$x_{m}=\sqrt{\frac{q}{16\pi {ɛ}_{Si}}\frac{W}{\left|V_{\text{Bias}}\right|}}\;\;\Delta \varphi_{B}=\sqrt{\frac{q}{4\pi {ɛ}_{\text{Si}}}\frac{\left|V_{\text{Bias}}\right|}{W}}$$

where ɛ_Si_ is the permittivity of silicon (10^−12^ C/cmV), W is the depletion width, and V_Bias_ the applied bias voltage.

Finally, the probability that an electron travels from the metal-semiconductor interface to the Schottky barrier maximum without scattering in the silicon is taken into account by the barrier collection efficiency η_c_, which can be written as [35]:

(4) $$\eta_{c}=e^{\frac{x_{m}}{L_{s}}}$$

where L_s_ is the electron scattering length in silicon. It is worth noting that by increasing the bias voltage, a shift of the Schottky barrier closer to the metal/semiconductor interface is obtained. In this way, barrier collection efficiency increases.

### 3.2. Two-Photon Absorption (TPA)

**Figure 4.**
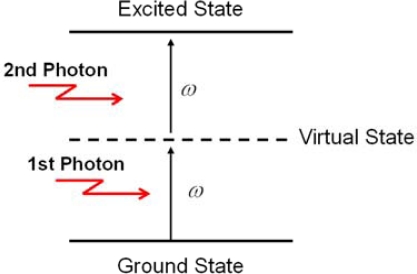
Two-photon absorption mechanism.

In the process of two-photon absorption, an electron absorbs two photons at approximately the same time, achieving an excited state that corresponds to the sum of the energy of the incident photons. In order to reach the final state, no intermediate state is necessary—only a “virtual state” which doesn’t correspond to any electronic or vibrational energy eigenstate (Figure 4).

The absorption cross section σ describing this process increases linearly with laser intensity according to the following relation:

(5) $$\sigma =\sigma^{\left(2\right)}I$$

where *σ*^(2)^ is a coefficient taking into account the probability that the two-photon absorption process occurs. It is clear that in conventional linear optics, the absorption cross section σ is a constant. Atomic transition rate R, due to two-photon absorption scales as the square of the laser intensity, is shown in the following formula:

(6) $$R=\frac{\sigma^{\left(2\right)}I^{2}}{\hbar \omega }$$

In the absence of significant recombination and diffusion, the differential equation describing the beam propagation in presence of linear and two-photon absorption can be written as [36]:

(7) $$dI/dz=-\alpha I-\beta I^{2}$$

where α is the linear absorption coefficient, β is the two-photon absorption coefficient, z is the longitudinal propagation direction, and where the instantaneous irradiance I = I(r, z, t) is a function of time t and transverse position r as well as z. By assuming α as constant, without taking into account the possible influence of lattice heating, band filling, and bandgap renormalization on α, Equation 7 can be solved exactly to give the transmitted irradiance I(r, L, t) in terms of the incident irradiance I(r, 0, t):

(8) $$I(r,\;L,\;t)=T_{0}\frac{I(r,\;0,\;t)}{1+\frac{I(r,\;0,\;t)}{I_{c2}}}$$

where:

(9) $$T_{0}=(1-R_{1})(1-R_{2})e^{-\alpha L}$$

is the linear transmission of a sample of thickness L and front (rear) surface reflectivity R_1_ (R_2_).

When the irradiance approaches the critical value I_c2_ defined as:

(10) $$I_{c2}=\frac{\left(\alpha /\beta \right)}{\left(1-R_{1})(1-e^{-\alpha L}\right)}$$

the transmission deviates from the linear value and TPA becomes comparable to linear absorption. It is worth noting that when I(r,0,t) = α/β, the two-photon coefficient near the surface equals the linear coefficient. Of course, (1 – R_1_) represents the front surface transmission while (1 − e^−αL^) is the linear absorption of a sample of thickness L. From a physics point of view, I(r,0,t)/I_c2_ compares the linear absorption coefficient α to the two-photon coefficient βI.

The quantity typically measured to derive β is the transmitted (T) energy, not the irradiance. For a Gaussian spatial and temporal irradiance profile, it can be shown that [36]:

(11) $$T^{-1}=T_{0}^{-1}\left[1-\frac{I_{0}}{2\sqrt{2}I_{c2}}\right]$$

It should be noted that this formula has been obtained without taking into account free-carrier absorption (FCA) generated by TPA. When FCA cannot be neglected, it can be included, obtaining a modified and more complex formula for the transmittance [36]. If front and back surface reflectivities are known, it is possible to show that the intercept of an inverse transmission *versus* irradiance plot corresponds to the linear absorption coefficient, while the initial slope is related to β.

**Figure 5.**
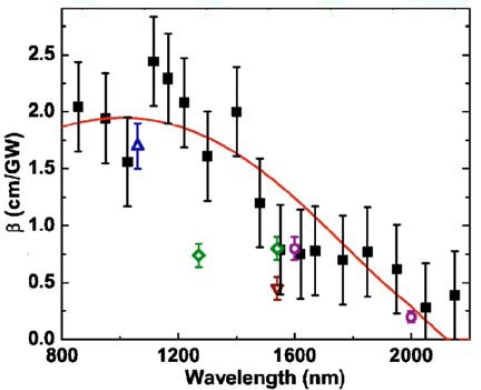
Measured value of β (squares) as a function of wavelength for a 125-μm-thick sample silicon wafer [43]. Data from other sources is also given: circles [44], up triangles [37], down triangles [39], and diamonds [41]. The solid curve represents the best fit based on calculations of Garcia and Kalyanaraman [45]. Reprinted with permission from A.D. Bristow, *et al.* [43]. (2007). Copyright 2007, American Institute of Physics.

Reintjes and McGroddy [37] isolated TPA in silicon for first time, obtaining a value of β = 1.5 cm/GW at 100 K for picosecond pulses at a wavelength λ = 1,060 nm, and, in 1986, Boggess *et al*. measured a similar value at 300 K [36]. In 1990, Reitze *et al*. reported 5 < β < 36 cm/GW by measurements across the direct gap of silicon in the range 550 < λ < 620 nm [38]. In recent years, TPA at telecommunications wavelengths of λ = 1,300 or λ = 1,500 nm has been reported, with values of β ∼ 0.8 cm/GW [39–41]. In 2007, Bristow *et al*. presented measurements for TPA across the indirect gap (Eg = 1.12 eV) at fundamental wavelengths between 850 and 2,200 nm and compared their results with theoretical predictions [42]. In their work, measurements of β are performed using a z-scan technique [43]. Values of β as a function of λ are illustrated in Figure 5. The data is compared with values measured by others.

## 4. All-Silicon Photodetector Structures

In this section, the main NIR silicon photodetector structures will be reviewed, taking advantage of the aforementioned physical effects.

### 4.1. MBA-Based Devices

**Figure 6.**
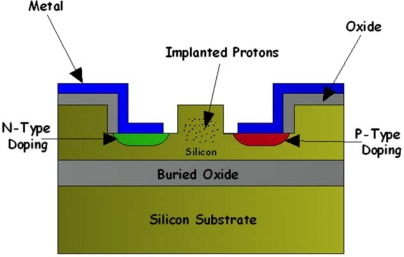
Monolithic p-i-n waveguide photodiode proposed by Knights *et al.* [46] in 2003.

MBA has been used to develop integrated optical detectors completely compatible with standard silicon technology and sensitive to C and L optical band wavelengths. Knights *et al.*, in a work for the Conference on Optical Fiber Communication in 2003, proposed a monolithic p-i-n waveguide photodiode based on implantation into a silicon-on-insulator wafer [46]. In particular, the implantation of protons into a rib waveguide with a lateral p-i-n diode (Figure 6) was used to create the deep levels. Thus, defects could be controlled in terms of concentration, depth and location via selective masking. On the other hand, shallow implantation and rapid thermal annealing of the p+ and n+ contact regions allowed for the separation of the contacts to be close to the width of the waveguide without introducing excessive free carrier absorption. The proposed 10-mm-long photodiode exhibited a responsivity of about 8 mA/W at 1,550 nm and a leakage current of about 3 nA, in both cases with 1 V of applied reverse bias.

**Figure 7.**
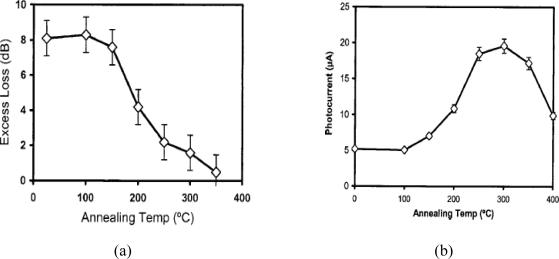
Optical loss **(a)** and photocurrent **(b)** *vs.* post-implantation annealing temperature for an ion-implanted integrated photodiode [48]. Reprinted with permission from A.P. Knights, *et al.* Copyright © 2006 IEEE.

Bradley, Jessop, and Knights have investigated different factors useful for improving both the performance and repeatability of integrated photodiodes [47,48]. Due to the high deep trap density, carriers are characterized by a lifetime in the nanosecond range and thus by a diffusion length of the order of a few microns. This implies a dependence of device performance on the separation between the waveguide, where the carriers are generated, and the semiconductor p-n junction [38]. Moreover, in 2006, Knights *et al*. [48] demonstrated the importance of post-implantation annealing on device performance. Figure 7(a,b) reports plots derived by the authors with regards to the dependence of the propagation optical losses and the photocurrent generated on the post-implantation annealing temperature for an integrated photodetector with defects introduced by silicon-ion bombardment. The plots demonstrate that an improvement of device performance can be obtained through low-temperature annealing (around 300 °C). For these temperatures, carrier recombination centers that do not contribute to the carrier generation process are removed. This should imply a reduction of the competing mechanisms of carrier generation and thus a significant improvement both of the photoresponse and optical loss within the waveguide.

Different implanted ions were used by Liu *et al*. [49]; in particular, helium ions were chosen for their non-reactivity with silicon and for stability of created defects [50]. Liu *et al*. carried out a detailed analysis of the influences of ion-implantation doses and of annealing temperature, both on the photogenerated current and the optical losses. This analysis led to the fabrication of an optimized integrated photodetector that was 1.7 cm long, and which obtained a maximum value of responsivity of 64 mA/W at 1,440 nm and a leakage current of 0.1 μA, both with a reverse bias of 20 V.

All the described photodetectors have been characterized by a very large traversal section in the order of tens of μm^2^, and by absorption lengths of 1 to 2 cm. Thus, the bandwidth of these devices was rather limited. Reducing the waveguide dimensions and optimizing the implantation in the silicon waveguide, Geis *et al.* [51] demonstrated the feasibility of robust high-frequency integrated photodiodes. The main concept is related to the decrease of the transversal cross section of the waveguide. In fact, this scaling induces a larger overlap between the optical mode and the implanted region and thus increases optical absorption, reducing the required device length and increasing the frequency response.

**Figure 8.**
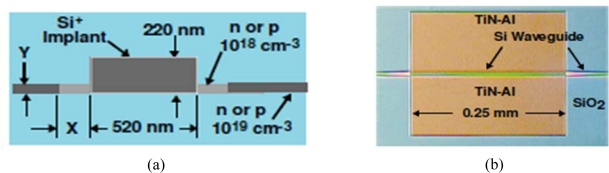
Top-view optical micrograph **(a)** and schematic cross section **(b)** of the integrated photodiode proposed by Geis *et al.* [53]. Copyright © 2009 Optical Society of America.

After a rigorous process of optimization [51,52], the photodetector proposed by Geis *et al.* had a cross section of about 0.11 μm^2^ showing an internal responsivity of 0.5–0.8 A/W at 1,550 nm, a reverse bias of about 5 V, a leakage current of 2.5 nA/mm and a bandwidth of 10–20 GHz. Further, to increase the device bandwidth, Geis and his co-authors optimized the external circuit components [53] such as the resistance and capacitance of the silicon wings on the sides of the rib wavelength (Figure 8) and on the contact pads.

**Figure 9.**
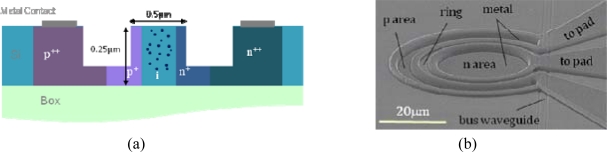
Cross section **(a)** and top view SEM image **(b)** of the fabricated ring detector [54]. Reprinted with permission from R. Shafiiha, *et al.,* Copyright © 2010 IEEE.

This optimization allowed minimizing the effect of the parasitic components and thus increased the detector bandwidth above 50 GHz. Unfortunately, Geis’s devices have low optical absorption, requiring a diode on the order of milimetres in length to absorb more than 50% of the incoming light. Recently, for the OFC/NFOEC 2010 conference in San Diego, Shafiiha *et al.* proposed a drastic reduction of the device size using a resonance structure [54]; in particular, the light from a straight waveguide coupled into a small-defect Si+ implanted ring resonator (Figure 9).

**Figure 10.**
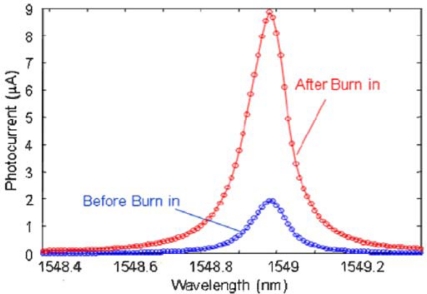
Photoresponse of a 15-μm-radius-ring detector at −2 V [54]. Reprinted with permission from R. Shafiiha, *et al.* Copyright © 2010 IEEE.

The ring resonator [55–57] was developed in order to equal the power coupling between the ring and the bus waveguide and the round-trip loss of the ring resonator at the resonance wavelength. In this condition, most of the transmission light is absorbed, increasing electron-hole generation. In Figure 9(a), the transverse section of the ring waveguide is sketched, and the overlap between the region of the optical mode, the defect area obtained and the depletion region of the p-i-n diode is illustrated. In Figure 9(b), a SEM image of the 15-μm-radius-ring integrated photodetector is reported. The photocurrent of the ring detector under a reverse bias of −2 V is illustrated in Figure 10.

In particular, photocurrents for both before and after a burn in process are reported. The burning-in process, that was also used by Geis *et al*. [52], consists of a forward bias with a current density of 300 mA/cm for 5 minutes and makes possible an increase in the responsivity of the ring photodetector from 0.02 A/W up to about 0.1 A/W at 1,549 nm and −2 V. Reported leakage current is 0.1 nA at −2 V of applied reverse bias. The compactness of the device allows reaching a 3-dB bandwidth of about 7 GHz at −2 V reverse bias. However, all the reported performances of the ring photodetector are relative to the resonance wavelength and thus the C+L bands are not completely covered.

To overcome this limitation, Doylend *et al*. recently proposed a new ring-resonator-based photodetector [58]. The device was characterized by a responsivity of 0.14 A/W with a reverse bias of −10 V and a leakage current of 0.2 nA; *i.e*., performance that is comparable with that of Shafiiha’s device. However, the novelty of the proposed structure is the possibility of tuning the photodetector response in a wavelength range from 1,510 nm to 1,600 nm. In particular, resonance wavelength shift has been obtained by thermo-optic tuning of the ring resonance, using a resistive metal strip which overlaps the structure.

**Figure 11.**
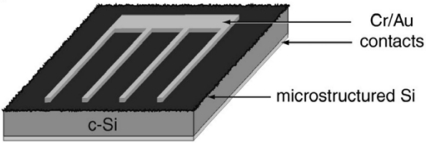
Bulk photodiode proposed by Carey *et al.* [60] in 2005. Copyright © 2005 Optical Society of America.

Although MBA has been well-known for quite some time, very few papers concerning silicon-based bulk devices have been reported in the literature, and yet several integrated devices are fabricated and characterized. In 2001, Wu *et al*. [59] observed a remarkable increase of IR absorbance in surface microstructured silicon wafers produced by laser irradiation in the presence of SF_6_. They found that in the microstructuring process, a high concentration of impurities and structural defects were incorporated into the silicon lattice, most likely producing bands of impurity states in the band gap that could absorb infrared radiation. The ion channelling spectra obtained from microstructured wafer show a background level higher than that of crystalline silicon but lower than that of amorphous silicon. These measurements indicate that the material is crystalline and is likely to have a high density of defects. The background remains high in ion channelling spectra from samples annealed for 3 hours at 1,200 K, indicating that a significant amount of disorder in the microstructured material is unaffected by annealing under these conditions. Moreover, the resulting subgap absorption is enhanced by the surface texture. These remarkable properties were then exploited for the fabrication of a silicon-based detector for infrared radiation [60]. The diode characteristics and responsivity depend strongly on processing conditions including laser fluence, substrate doping, and thermal annealing temperature. Figure 11 shows a schematic diagram of the device, whose active area is approximately 5 mm^2^.

This optimized sample exhibits responsivity of 50 mA/W at 1,330 nm and 35 mA/W at 1,550 nm at 0.5 V of applied reverse bias; its capacitance is 64 nF/cm^2^, while the signal rise time is 10 ns and the fall time is 30 ns. Device leakage current is 120 μA/cm^2^ at 0.5 V of applied reverse bias.

### 4.2. SSA-Based Devices

**Figure 12.**
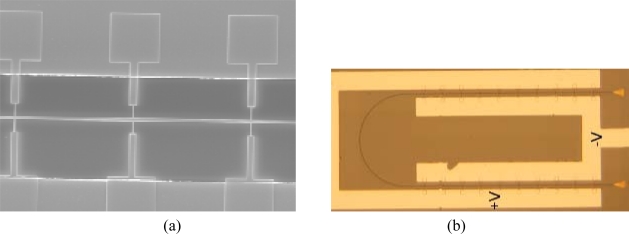
Top view SEM image **(a)** and optical image **(b)** of the detector proposed by Baehr-Jones *et al.* [61]. Copyright © 2008 Optical Society of America.

In 2007, Geis *et al.* [51] observed a low photoresponse for an unimplanted diode and attributed it to a surface effect of the waveguide even if its performances were a strong function of the operating environment (such as humidity and surface chemistry). Baehr-Jones *et al*. [61] enhanced the effect of the surface states optimizing the overlap between the optical mode and the surface of the waveguide. The SEM image and a top view of one of the devices characterized by the authors are shown in Figures 12(a,b), respectively.

Small conductions arms intersects the waveguide defining the active regions of the photodetector. The waveguide was developed with a cross section of 500 nm × 100 nm in order to increase overlap between the optical mode and the waveguide surface. In this way, part of the optical mode is absorbed by the surface states and a change in conductivity of the device is achieved. For the device illustrated in Figure 12, a responsivity of 36 mA/W for a bias of 11 V at a wavelength of 1,575 nm was measured. A device leakage current of 0.12 μA at −11 V is reported as well. Moreover, the authors reported a detailed analysis to demonstrate that the measured photocurrent is due to the energy state of the surface defects and not to other effects such as heating, two-photon absorption or internal photoemission.

**Figure 13.**
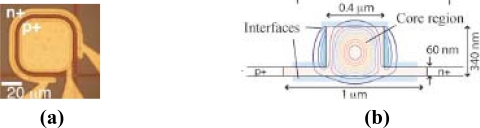
**(a)** Top view of the ring resonator detector. **(b)** Sketch of the device and contour of optical mode [62]. Reprinted with permission from H. Chen, *et al.* Copyright 2009, American Institute of Physics.

In order to enhance the photocurrent generated by SSA, Chen *et al.* [62] used a p-i-n diode embedded into a silicon micro-ring resonator. Figure 13(a) shows a top view of Chen’s proposed device; whereas in Figure 13(b), the waveguide dimension obtained on a SOI wafer with a 1-μm-thick buried oxide layer is illustrated. A low-temperature deposited oxide layer with a thickness of 1 μm has been used to cover the waveguide. In Figure 13(b), the intensity contour of the simulated TE optical mode field is reported and the spatial overlapping with the Si/SiO_2_ interfaces can be noted. This overlapping makes possible achievement of photogenerated current by SSA.

The ring resonator was characterized by a Q factor of about 8,000. With this value, the responsivity detected at the resonance wavelength (1,541.5 nm) for 0 V bias was about 0.12 mA/W and resulted twenty times that of the responsivity measured for off-resonance wavelengths. Increasing the bias voltage to −15 V, the responsivity increased to 0.25 mA/W but the dark current reached 2.5 nA. Due to the presence of a resonator, the device became more sensitive to temperature changes. In particular, the authors measured a redshift of the resonance wavelength that was explained by the thermo-optical effect and the heating due to the carrier recombination. Finally, the authors noted a slight linear absorption-induced photovoltaic effect, with a power generation efficiency of about 0.05 mW/W.

### 4.3. IPA-Based Devices

**Figure 14.**
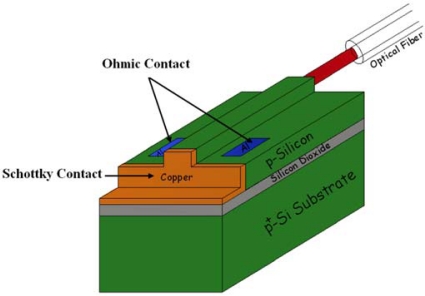
Schematic view of the Cu/p-Si Schottky-barrier-based integrated photodetector proposed by Casalino *et al.* [63]. Reprinted with permission from M. Casalino, *et al*. Copyright 2010, American Institute of Physics.

For all the previous described methods, a photon at a wavelength of 1.55 um that travels in the silicon waveguide is efficiently absorbed if its energy allows an inter- or intra-band transition of an electron (or hole). Another way to absorb the photon is to lower the energy gap that the electrons (holes) must overcome. This approach is based on the use of a semiconductor/metal interface. The potentialities of such a method for the realization of an all-silicon photodetector have been demonstrated by the theoretical work of Casalino *et al.* [29], and recently the effect has been employed for an integrated structure as well [63]. In Figure 14, the integrated photodetector proposed by Casalino *et al.* [63] is schematically illustrated. A rib waveguide was terminated on a deep trench that reached down to the buried oxide layer of the SOI wafer. A Cu/p-Si Schottky contact was fabricated on the vertical surface of the deep trench.

**Figure 15.**
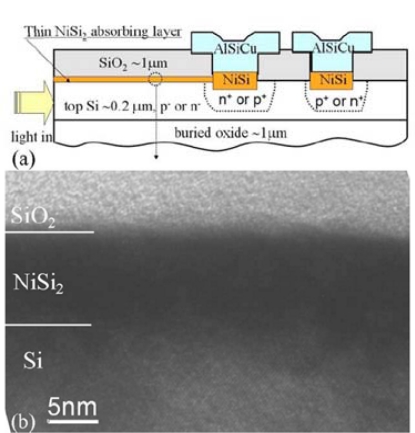
**(a)** Schematic structure of the waveguide-based silicide Schottky-barrier photodetector proposed by Zhu *et al.*; **(b)** XTEM of the thin absorbing NiSi_2_ layer on the silicon waveguide [64]. Reprinted with permission from S. Zhu, *et al*. Copyright 2008, American Institute of Physics.

By means of this technological solution, a very narrow semiconductor/metal barrier transverse to the optical field coming out from the waveguide has been achieved. The integrated photodetector was characterized by a responsivity of 0.08 mA/W at a wavelength of 1,550 nm with a reverse bias of −1 V. Measured dark current at −1 V was about 10 nA. Moreover, the authors assert that the thinness of the Cu/p-Si Schottky barrier could enable a speed operation in the gigahertz range. An indirect evaluation of the bandwidth of the detector was reported to confirm the operation speed potentialities. A bandwidth of about 3 GHz was measured by Zhu *et al.* [64] on a Schottky-barrier-based integrated photodetector, where the junction was achieved by a nickel silicide layer (NiSi_2_) on silicon [65,66] (Figure 15).

In order to achieve both a suitable optical absorption and an efficient photoexcitation of metal electrons or holes across the silicide/silicon interface, the authors proposed the lengthening of a thin silicide layer on the surface of a SOI waveguide. The authors reported a detailed analysis on the influence of the silicide layer dimension on the performance of both NiSi_2_/p-Si and NiSi_2_/n-Si diodes. The overall performance of the NiSi_2_/p-Si structure has resulted better than that relative to the NiSi_2_/n-Si interface, due to the lower Schottky barrier height of the NiSi_2_/p-Si structure. In particular, a responsivity of about 4.6 mA/W at a wavelength of 1,550 nm and a reverse bias of −1 V was estimated for the NiSi_2_/p-Si diode against the value of 2.3 mA/W of the NiSi_2_/n-Si junction. Moreover, a measured dark current of 3 nA was reported.

Recently, Akbari *et al.* [67] have proposed enhancing the interaction between the optical field and the metal/semiconductor barrier by the use of surface plasmon polariton (SPP) [68,69]; *i.e*., an optical surface wave propagating along the interface between a metal and a dielectric [70]. The performances achieved are interesting and comparable with other Schottky-barrier-based integrated photodetectors. In particular, the authors measured a responsivity of about 0.8 mA/W at a wavelength of 1,550 nm and a bias voltage of −100 mV for a 35-μm-long SPP detector obtained by an Al strip on n-Si; while the dark current was about 6 μA.

**Figure 16.**
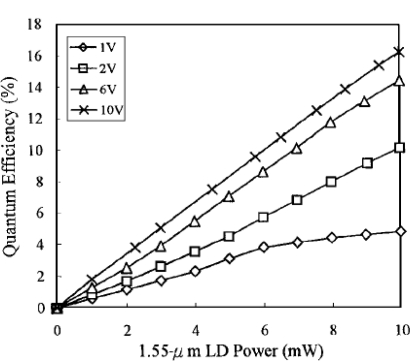
Photoconductive gain *versus* illumination power of a 1.55-μm laser diode with the bias voltage as a parameter for the photodetector proposed by Lee *et al.* [71] in 2001. Copyright © 2001 Optical Society of America.

Recently, some research groups have published papers on IPA-based NIR silicon bulk photodetectors working at room temperature. Lee *et al.* [71] investigated the spectral responsivity of Al-porous silicon Schottky barrier photodetectors in the wavelength range 0.4–1.7 μm. The structure of the PS photodetector was Al (finger type)/PS/Si/Al (ohmic), and the active area was 18 mm^2^. The photodetectors show strong photoresponsivity in both the visible and the infrared bands, especially at 1.55 μm. The photocurrent can reach 1.8 mA at a reverse bias of 6 V under illumination by a 1.55-μm, 10-mW laser diode. The corresponding quantum efficiency is 14.4%; this high value comes from a very high surface-area-to-volume ratio, of the order of 200–800 m^2^/cm^3^ of porous silicon. The dark current is −5 μA at −10 V. The quantum efficiency *versus* illumination power under 1.55 μm semiconductor laser diode illumination with the bias voltage as a parameter is shown in Figure 16. The quantum efficiency is almost a linear function of the illumination power. In addition, the quantum efficiency increases and gradually saturates with reverse-bias voltage. The frequency response was 200 MHz and is limited by the low carrier mobility.

**Figure 17.**
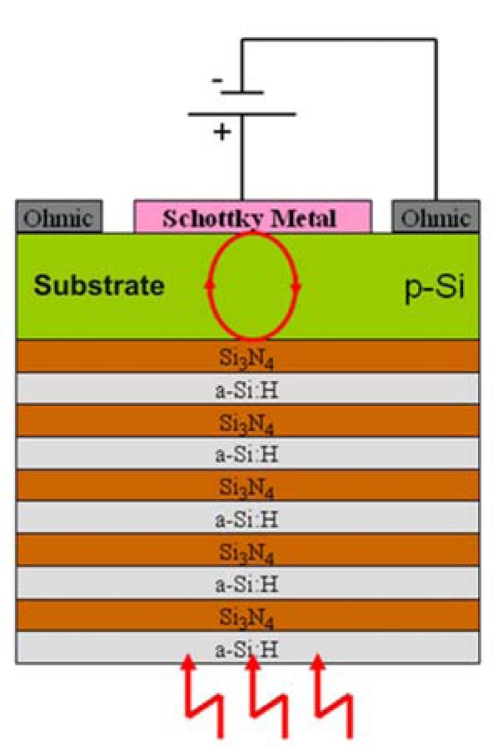
Schematic cross section of the photodetector proposed by Casalino *et al.* [72,73]. Reprinted with permission from M. Casalino *et al.* Copyright © 2010 IEEE.

More recently Casalino *et al.* [72,73] presented the realization and the characterization of a resonant cavity enhanced (RCE) photodetector, completely silicon compatible and working at 1.55 μm (see Figure 17). The resonant cavity is a vertical-to-the-surface Fabry-Perot structure. It is formed by a buried reflector, a top mirror interface and, in the middle, a silicon cavity. The buried reflector is a Bragg mirror, realized by alternating layers of amorphous hydrogenated silicon (a–Si:H) and silicon nitride (Si_3_N_4_). A Schottky metal layer (Cu), working both as active (absorbing) layer and as cavity mirror, is deposited above the silicon layer. The novelty of the device is the idea of enhancing the internal photoemission absorption by the microcavity effect. The room temperature responsivity measurements on the device reveal a peak responsivity of about 2.3 μA/W and 4.3 μA/W, respectively, for 0 V and −10 mV of applied reverse bias for a device having radius of active area of 2 mm [72]. Recently the same authors have improved device performances by reducing the radius of active area down to 40 μm obtaining a responsivity of 8 μA/W at −100 mV. In this device a bandwidth in the GHz range was estimated, too [73].

### 4.4. TPA-Based Devices

**Figure 18.**
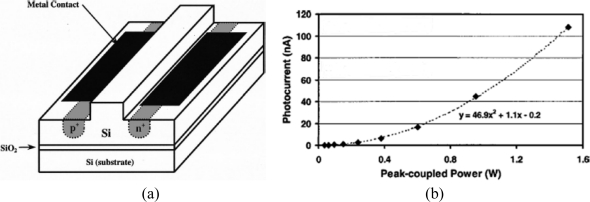
Sketch **(a)** and measured photocurrent **(b)** of the TPA-based detector proposed by Liang *et al.* [75]. Reprinted with permission from T.K. Liang, *et al.*, Copyright 2002, American Institute of Physics.

Despite the small value of the TPA coefficient, ranging from 0.44 cm/GW to 0.9 cm/GW at a wavelength of 1,550 nm [74], silicon optical waveguides have potential for use in TPA because of their long interaction lengths and low optical dispersion in silicon. The photocurrent generated by the TPA effect can efficiently collect embedding a p-i-n diode in the silicon waveguide. In Figure 18, the TPA-based integrated detector proposed by Liang *et al*. in 2002 is reported [75].

**Figure 19.**
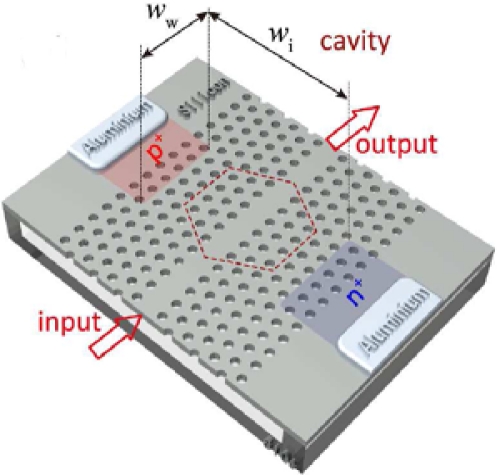
Schematic view of the device proposed by Tanabe *et al.* [77]. Reprinted with permission from T. Tanabe, *et al.* Copyright 2010, American Institute of Physics.

In Figure 18, the measured photocurrent for a bias voltage of −3 V is reported and the typical non-linear behaviour of the TPA effect can be noted. Liang’s device was used to perform an optical autocorrelation for measuring ultra-short laser pulses, but it was not suitable as a photodetector due to the high required optical input power value. The key to obtaining micrometer devices sensitive to low optical input is to strengthen the interaction between light and matter by tightly confining the light, *i.e.*, employing high-Q microresonators. Bravo-Abad *et al.* [76] theoretically studied the feasibility of using p-i-n diode embedded silicon micro-ring or photonic crystal resonators for ultrafast photodetection. Starting from these premises, Tanabe *et al.* [77] proposed an infra-red photodetector based on a p-i-n integrated into a high-Q width-modulated line-defect photonic crystal (PhC) nanocavity [78,79]. The sketch of the device proposed by the Japanese authors is reported in Figure 19. The nanocavity is characterized by a lattice constant *a* = 420 nm, hole radius *r* = 108 nm, slab thickness *t* = 204 nm, *Wi* = 8.72 μm and *Ww* = 8.4 μm; whereas the input and output waveguides are 1.05*a* wide and are in-line connected with the cavity through barrier line defects 0.98*a* wide and 11*a* long.

The PhC nanocavity is characterized by an intrinsic Q factor of 8.4 × 10^5^. This high Q value permits the obtention of a high generation of photocurrent into the cavity region, enabling the possibility of detecting even low input power. In particular, an external quantum responsivity of 16 mA/W at a resonance wavelength of aproximately 1,500 nm, has been measured. Furthermore, on the one hand, the very small dimensions of the device allow achievement of a low dark current value (about 15 pA at room temperature for 3 V of reverse bias), and on the other hand, a small capacitance (a value of 9.5 × 10^−18^ F was estimated). The resistance for the p and n regions limits the speed of the proposed device at 0.1 GHz.

A silicon microdisk resonator with a laterally embedded p-i-n diode was chosen by Chen *et al.* [80] to enhance the TPA photocurrent. TPA is a very weak effect which is difficult to exploit for the realization of bulk all-silicon photodetectors with appreciable efficiency. For this reason, as shown in the literature, TPA exploitation in bulk silicon devices at 1,550 nm is aimed at improving the sensitivity of two-photon absorption autocorrelators, utilizing, as the two-photon absorber, a silicon avalanche photodiode (APD) [81–83]. The avalanche process in APDs amplifies the weak IR two-photon absorption photocurrent, thereby greatly improving the sensitivity of the autocorrelation measurement. More recently, TPA in all-silicon bulk detectors has been exploited for a novel profilometry technique at λ = 1.55 μm [84]; compared with conventional profilometry, TPA has a much wider dynamic range without the need for high-speed devices or complicated computation. Shi *et al.* [85] fabricated a hemispherical nearly-intrinsic silicon TPA photodetector operating at a wavelength of 1.3 μm and emitted from a CW laser. The detector was made of nearly-intrinsic silicon crystal with a resistivity of 6,000 Ω/cm and made into a hemisphere with a radius of 3 mm. The silicon hemisphere was used as both a detector and a solid immersion lens (SIL) in the experiments. The bottom of the detector is (1̅10) plane, on which aluminum electrodes were evaporated. The electrodes consisted of concentric circular and annular metal contacts with a spacing of 0.15 mm and a radius of 0.5 mm for the central electrode. The contacts between the aluminum and the silicon hemisphere are considered to be ohmic contacts. Because TPA substantially occurs in the vicinity of the focused spot (the center of the hemisphere), the concentric electrodes collect the photo-excited carriers efficiently.

The responsivity of the detector was about 2 μA/W at 1,300 nm and 1 V of applied bias. This silicon photodetector is easy to fabricate and is useful in autocorrelation for measuring ultra-short laser pulses with wavelengths in the region of 1.2–2.1 μm.

## 5. Conclusions

In this work an overview on the NIR all-Si photodetectors has been presented. First, we have attempted to elucidate the most utilized physical effects allowing Si absorption at sub band-gap wavelength such as: mid-bandgap absorption, surface state absorption, internal photoemission absorption and two photon absorption. Then a quantitative comparison of the photodetectors proposed in the scientific literature based on the aforementioned absorption mechanisms, including both bulk and integrated devices, have been reviewed and summarized in Table 1. Overall, MBA based waveguide photodetectors seem to show the best performances if compared to devices based on others absorption mechanisms. In recent years a drastic reduction of the NIR all-Si photodetector size has been the most urgent task to reach, for this reason new structures such as MBA and SSA based ring resonator photodetectors and TPA based photonic crystal nanocavity photodetectors, have been fabricated.

The scaling down of the devices, however, is still in progress and new perspectives have been opened by the surface plasmon polaritons (SPP) and its demonstrated capability to guide electromagnetic energy below the diffraction limit. It is our opinion that IPA based photodetectors associated to plasmonic waveguiding structures could play a key role in the future of the NIR all-Si photodetection and could be the new frontier in the field of low-cost silicon photonics.

## Figures and Tables

**Table 1.** Performance summarization of NIR all-silicon photodetectors reported in the literature.

**Responsivity**	**Dark/leakage current**	**Bandwidth**	**Dimension**	**Effect**	**Type**	**Ref.**
8 mA/W (λ = 1,550 nm V_bias_ = −1 V)	∼ 3 nA (V_bias_ = −1 V)	-	10 mm (waveguide length)	MBA Proton implantation	Integrated	[46]
64 mA/W (λ = 1,440 nm V_bias_ = −20 V)	0.1 μA (V_bias_ = −20 V)	-	1.7 cm (waveguide length)	MBA Helium ion implantation	Integrated	[49]
0.5 – 0.8 A/W (λ = 1,550 nm V_bias_ = −5 V)	2,5 nA/mm (V_bias_ = −5 V)	10–20 GHz	0,11 μm^2^ (cross section) Waveguide length on the order of mm	MBA Si^+^ implantation	Integrated	[51,52]
0.1A/W (λ = 1,549 nm V_bias_ = −2 V)	0.1 nA (V_bias_ = −2 V)	7 GHz	15 μm (radius of ring resonator)	MBA Si^+^ implantation ring resonator	Integrated	[54]
50 mA/W (λ = 1,330 nm V_bias_ = −0.5 V) 35 mA/W (λ = 550 nm V_bias_ = −0.5 V)	120 μA/cm^2^ (V_bias_ = −0.5 V)	-	5 mm^2^ (active area)	MBA Laser irradiation in presence of SF_6_	Bulk	[60]
36 mA/W (λ = 1,575 nm V_bias_ = −11 V)	0.12 μA (V_bias_ = −11 V)	-	500 nm × 100 nm (cross section)	SSA	Integrated	[61]
0.25 mA/W (λ = 1,541.5 nm V_bias_ = −15 V)	2.5 nA (V_bias_ = −15 V)	-	-	SSA-ring resonator	Integrated	[62]
6 mA/W (Around λ = 1,550nm V_bias_ = −3 V)	15 pA (Vbias = −3 V)	-	-	TPA Photonic crystal resonators	Integrated	[77]
2 μA/W at (λ = 1,300 nm V_bias_ = 1 V)	-	-	3 mm (hemisphere radius)	TPA Hemispherical structure	Bulk	[85]
0.08 mA/W (λ = 1,550 nm V_bias_ = −1 V)	10 nA for (Vbias = −1 V)	Estimated GHz range	-	IPA Cu/p-Si Schottky barrier	Integrated	[63]
4,6 mA/W (λ = 1,550 nm V_bias_ = −1 V)	3 nA (V_bias_ = −1 V)	3 GHz	-	IPA NiSi_2_/p-Si Schottky barrier	Integrated	[64]
0,8 mA/W (λ = 1,550 nm V_bias_ = −100 mV)	6 μA (V_bias_ = − 100 mV)	-	35 μm (stripe length)	IPA Surface plasmon polariton	Integrated	[67]
0,18 A/W (λ = 1,550 nm V_bias_ = −6 V)	5 μA (V_bias_ = −10 V)	-	18 mm^2^ (active area)	IPA Al-porous Si Schottky barrier	Bulk	[71]
8 μA/W (Around λ = 1,550 nm Vbias = −100 mV)	-	Estimated GHz range	40 μm (radius of active area)	IPA Cu/p-Si Schottky barrier	Bulk	[73]
